# A Breast Tumor Monitoring Vest with Flexible UWB Antennas—A Proof-of-Concept Study Using Realistic Breast Phantoms

**DOI:** 10.3390/mi15091153

**Published:** 2024-09-14

**Authors:** Rakshita Dessai, Daljeet Singh, Marko Sonkki, Jarmo Reponen, Teemu Myllylä, Sami Myllymäki, Mariella Särestöniemi

**Affiliations:** 1 Microelectronics Research Unit, University of Oulu, 90014 Oulu, Finlandsami.myllymaki@oulu.fi (S.M.); 2 Health Sciences and Technology, Faculty of Medicine, University of Oulu, 90014 Oulu, Finland; daljeet.singh@oulu.fi (D.S.); jarmo.reponen@oulu.fi (J.R.); teemu.myllyla@oulu.fi (T.M.); 3 InfoTech Oulu, University of Oulu, 90014 Oulu, Finland; 4 Ericsson Antenna Technology Germany GmbH, 83026 Rosenheim, Germany; marko.sonkki@gmail.com; 5 Medical Research Centre, Oulu University Hospital, University of Oulu, 90220 Oulu, Finland; 6 Optoelectronics and Measurements Research Unit, Faculty of Information Technology and Electrical Engineering, University of Oulu, 90570 Oulu, Finland; 7 Centre for Wireless Communications, Faculty of Information Technology and Electrical Engineering, University of Oulu, 90570 Oulu, Finland

**Keywords:** antenna measurements, breast cancer, biodevices, biomedical monitoring, biosensors, dielectric properties, healthcare technology, microwave diagnosis

## Abstract

Breast cancers can appear and progress rapidly, necessitating more frequent monitoring outside of hospital settings to significantly reduce mortality rates. Recently, there has been considerable interest in developing techniques for portable, user-friendly, and low-cost breast tumor monitoring applications, enabling frequent and cost-efficient examinations. Microwave technique-based breast cancer detection, which is based on differential dielectric properties of malignant and healthy tissues, is regarded as a promising solution for cost-effective breast tumor monitoring. This paper presents the development process of the first proof-of-concept of a breast tumor monitoring vest which is based on the microwave technique. Two unique vests are designed and evaluated on realistic 3D human tissue phantoms having different breast density types. Additionally, the measured results are verified using simulations carried out on anatomically realistic voxel models of the electromagnetic simulations. The radio channel characteristics are evaluated and analyzed between the antennas embedded in the vest in tumor cases and reference cases. Both measurements and simulation results show that the proposed vest can detect tumors even if only 1 cm in diameter. Additionally, simulation results show detectability with 0.5 cm tumors. It is observed that the detectability of breast tumors depends on the frequency, antenna selection, size of the tumors, and breast types, causing differences of 0.5–30 dB in channel responses between the tumorous and reference cases. Due to simplicity and cost-efficiency, the proposed channel analysis-based breast monitoring vests can be used for breast health checks in smaller healthcare centers and for user-friendly home monitoring which can prove beneficial in rural areas and developing countries.

## 1. Introduction

Breast cancer is a universal health concern of paramount significance which affects millions of women across the globe. Breast cancer resulted in 2.3 million diagnoses and more than 685,000 deaths in 2020 [1,2]. Early detection is critical but challenging for the successful treatment of breast cancer as almost half of the subjects affected by breast cancer do not show any visually detectable risks in the early stages or family history. The current methods for breast cancer detection include mammography screening, 3D mammography, breast Magnetic Resonance Imaging (MRI), breast ultrasound, and clinical breast examination (CBE) which have certain limitations in terms of expensive large-sized equipment, special resources, patient comfort during screening procedures, and the requirement of trained staff [3]. These limitations require typical healthcare service settings and hinder the prospects of home monitoring by the subject itself or the widespread adoption of these devices outside of hospital settings due to poor portability. Furthermore, the accurate interpretation of screening results requires expertise, and false positive and false negative results can cause unnecessary anxiety or unnoticed cases of cancer, respectively [3]. 

To address these limitations and challenges, researchers have explored the use of microwave technology for breast cancer monitoring extensively in [4,5,6,7,8,9,10,11,12,13,14,15,16,17,18,19,20,21,22,23,24,25,26,27,28,29,30,31]. Microwave technology works on the principle of differentiating cancerous tissue from healthy breast tissue based on its distinct dielectric properties, enabling the detection of cancerous tissue through radio channel responses between multiple antennas placed around the breast [6,32]. Optimized antenna configuration is utilized to capture the differences in dielectric properties which can be further analyzed using artificial intelligence (AI)-based approaches on server computers. 

Microwave-based breast cancer detection presents several advantages over traditional methods such as mammography, ultrasound, and MRI, particularly in terms of efficiency, cost, and accessibility. Microwave imaging can generate high-resolution images and detect tumors at early stages by leveraging the differential dielectric properties of tissues to identify abnormalities, which is especially beneficial for dense breast tissues. Additionally, microwave systems are generally less expensive to manufacture and maintain compared to MRI machines and mammography equipment, making them a more cost-effective option for widespread screening. Furthermore, due to their lower cost and portability, microwave systems have potential to be more readily deployed in remote or underserved areas, thereby increasing accessibility for populations that may not have regular access to advanced medical facilities.

Different breast tumor detection systems with ultrawideband (UWB) antennas are proposed in the literature [12,13,14,15,16,17,18,19,20,21,22,23,24,25,26,27,28,29,30,31]. These designs utilize UWB antenna arrays for breast cancer detection, showcasing their potential in improving the early detection of small-sized tumors up to 1 cm. Klemm et al. [12] developed a UWB microwave imaging system operating at 4.5–10 GHz using cavity-backed patch antennas and a 3D hemispherical antenna array with 16 UWB aperture-coupled stacked patch antennas. Meaney et al. [13] created a clinical prototype for active microwave imaging of the breast, employing a 32-channel data acquisition system with 16 circularly arranged monopole antennas operating at 300 MHz–1 GHz.

Seong-Ho et al. [14] modified the system from [12] to develop a microwave tomography system operating at 500 MHz–3 GHz, featuring vertical antenna adjustment and a high-sensitivity transmitter. Amineh et al. [15] introduced a near-field microwave imaging system using UWB antennas and a raster scan algorithm, tested on 3D breast phantoms. Flores et al. [16] conducted a preclinical study using a single-element Vivaldi antenna for cylindrical-shaped dielectric objects.

Mohammed et al. [17] proposed a microwave imaging system with a 12-element Tapered Slot Antenna (TSA) configuration for breast imaging. Bahramiabarghouei et al. [18] designed a UWB breast cancer detection system for a bra, tested on a rubber-based phantom model. Rahman et al. [19] proposed a bi-static radar-based breast imaging system using flexible UWB antennas, tested on a homogeneous phantom model. Another system [20] used vertically and horizontally polarized antennas to reduce SAR values, operating at 4.8–30 GHz.

Phasukkit et al. [21] presented an impulse radio-based UWB antenna setup for breast tumor localization, using rigid Rogers RT/duroid 5870 material. Jafarifarmand et al. [22] proposed a Neural Network (NN)-based breast imaging system, reducing features by a factor of seven. Lu et al. [23] introduced a framework using CNN and LSTM for breast cancer localization. A textile monopole antenna-based system for breast tumor monitoring was presented in [24], incorporating a machine learning algorithm for processing S21 data.

The authors’ previous study [29] proposed a simulation-based approach using UWB flexible antennas embedded in self-monitoring vest to detect early-stage small breast tumors (1 cm) deep inside the glandular tissue where breast tumor usually starts to grow. This was the first study presenting breast tumor detection results with anatomically realistic simulation model having realistic glandular and adipose tissue constitution (i.e., not only layer model simulations). Ref. [29] was also the first study to propose a self-monitoring vest that ensures it is properly worn to avoid uncertainties in measurement results. In general, wearable sensor-based monitoring systems can encounter challenges due to variations in sensor placements. Consequently, it is essential to design wearable systems to minimize potential uncertainties arising from how the device is worn. The authors have also developed an initial proof-of-concept for the breast tumor monitoring vest [30,31], in which the cabling is designed to avoid these uncertainties in the measurement results. Additionally, this vest was evaluated for the first time with realistic breast tissue phantoms having different breast densities and sizes [10]. 

This paper is an extension of the work presented in [30]: the paper describes the development process of the monitoring vest thoroughly and presents more comprehensive evaluations for tumor detection with simulations and measurements. The monitoring vest setup is optimized using three different types of flexible and textile antennas operating in the UWB frequency range 3.1–10.6 GHz and Industrial Scientific and Medical (ISM) 2.45 GHz band. Realistic 3D breast tissue-mimicking phantoms for different breast types and sizes are utilized for evaluating the performance of the proposed setup. The realistic phantom consists of skin, muscle, fat, tumor, and glandular tissues that accurately replicate the dielectric properties of real tissues within the desired frequency range [10,32]. Additionally, the phantoms maintain stable electromagnetic properties over time and are produced using cost-effective and non-toxic materials which smoothens the preservation process. Based on numerous trials made during measurement results, it is observed that the proposed vest does not require any clinical expertise for its operation and can be easily worn by the subject any time for breast cancer self-monitoring as well. 

The proposed vest is a computationally simple biodevice that analyzes channel parameters between various flexible antennas. In practical applications, the measured data would be compared with reference data specific to the individual’s breast type, as detailed in [9]. These reference data banks are categorized by different breast sizes and density types [9]. The vest can indicate the potential presence of tumors and recommend that the individual visit a hospital for more detailed imaging. An advanced version of the vest could generate images from the measured channel parameters using various imaging algorithms [7]. However, this would necessitate extensive computational resources, thereby increasing the cost of the monitoring vest and limiting its practicality.

This work focuses on the description and evaluation of the first proof-of-concept of the breast tumor monitoring vest, which incorporates flexible UWB antennas. The primary aim is to detail the development of the initial proof-of-concept versions of the monitoring vests and present evaluation results using realistic models for different breast densities.

Relative to the prior studies utilizing microwave technique-based vests for breast cancer monitoring, this paper introduces several innovative components:

1. The vest has undergone assessment with a variety of flexible antennas, all of which are deemed suitable for real-world application;
2. Cabling of the vest is planned to avoid uncertainties in the measurement results and enabling also self-monitoring;
3. The evaluations have been conducted using realistic phantoms that vary in size and breast density, alongside accurate and realistic simulation models;
4. Furthermore, the effectiveness of tumor detection has been tested using models where the tumor is situated deep within the glandular tissue where tumors start to grow. This presents a greater challenge compared to detecting tumors within fatty tissue, as the dielectric property differences between tumors and glandular tissue are significantly less pronounced than those between tumors and fatty tissue;
5. This is the first paper presenting detectability of 0.5 cm sized tumors with flexible antennas and anatomically realistic simulation models.

This paper is organized as follows: Section 2 presents Methods and Procedures, including a description of the designed phantoms, flexible and textile antennas for monitoring vests, and different vest versions. Results and Discussions are presented in Section 3 for measurements with different vest versions along with different sets of antennas. Additionally, simulation results with realistic voxel modes are presented for validation of measurement results. Finally, Conclusions are given in Section 4.

## 2. Methods and Procedures

### 2.1. Principle of Breast Cancer Detection with Microwaves

The female breast can be anatomically divided into two main tissue types: adipose tissue, primarily composed of fat, and fibro glandular tissue, consisting of glands and connective tissue. The Breast Imaging Reporting and Data System (BI-RADS) [33] categorizes breasts into four classes based on their fibro glandular density: Class I, fatty breasts; Class II, scattered fibro glandular density; Class III, heterogeneously dense breasts; and Class IV, extremely dense breasts. Breast density plays a significant role in tumor detection, as dense breasts can pose challenges in distinguishing tumors from normal glandular tissue during traditional mammography. Human breast tissue exhibits specific microwave properties that can be characterized by its dielectric properties, including relative permittivity and conductivity. These properties play a crucial role in the interaction of microwave radiation with breast tissue during imaging and detection processes. The relative permittivity of breast tissue varies with the frequency of the applied microwave radiation [32].

At higher frequencies (microwaves), the dielectric properties are primarily influenced by the water content in the tissue, while at lower frequencies (radiofrequency and below), the contribution of other constituents such as membranes and proteins becomes more significant. The outermost layer of the breast is the skin, which has relatively high dielectric properties compared to adipose, i.e., fat tissue. Most of the breast is composed of adipose tissue which has low relative permittivity and conductivity, primarily due to its low water content. Microwave radiation tends to propagate through adipose tissue with minimal absorption and attenuation. Another layer in the breast is the fibro glandular tissue, which contains glands, ducts, and connective tissue. This tissue has a higher water content compared to adipose tissue, resulting in higher relative permittivity and conductivity. Microwave radiation experiences increased absorption and attenuation when passing through fibro-glandular tissue. The detection of abnormalities or anomalies in the breast using microwave imaging techniques relies on the differences in the dielectric properties of healthy tissue and pathological conditions, such as tumors or lesions. When a radio signal hits the interface between two tissues or materials with different dielectric properties, it can be reflected, scattered, or transmitted. These signal components can combine positively or negatively at the receiving antenna. Factors such as the antenna’s frequency, polarization, radiation pattern, and placement on the skin also influence signal propagation through tissues. These variations in signal propagation near a tumor can be detected by analyzing antenna reflection coefficients (S11) and channel parameters (S21, S31, S*N1*, where *N* is the number of antennas). 

### 2.2. Phantoms

**Figure 1:**
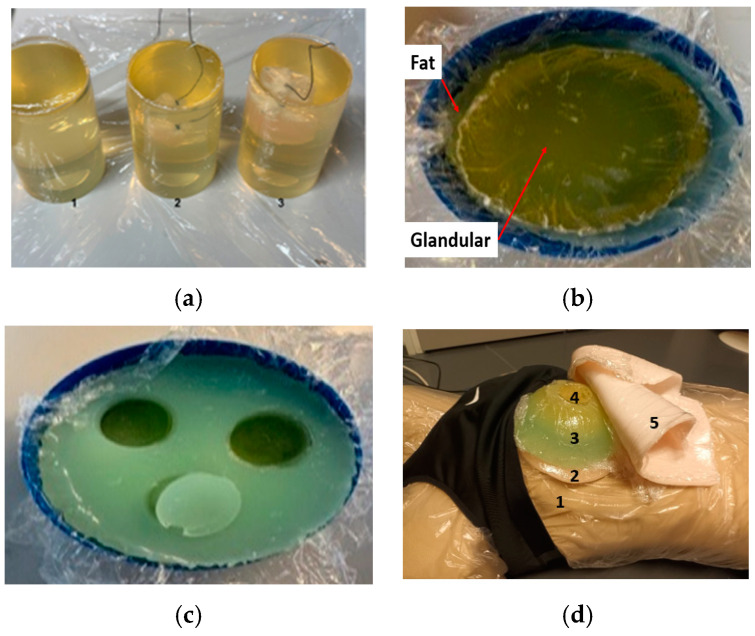
(**a**) Three cylindrical-shaped glandular phantoms: (1) reference, (2) with 1 cm tumor, and (3) with a 2 cm tumor; (**b**) breast phantom “Very Dense” with 0.5 cm thick fat layer; (**c**) breast phantom “Dense” with the glandular phantom inserted into the fat phantom; (**d**) measurement setup with phantoms set on the mannequin torso (1), above which the muscle phantom is first assembled (2), fat (3), glandular (4), and skin (5) phantoms [30].

For this study, multilayer breast tissue phantoms, i.e., fat, breast glandular, skin, tumor, and muscle, were developed using gelatin-based recipes. A detailed description of the phantom development and procedure can be found in [10]. Table 1 summarizes the recipes used for phantom fabrication. The dielectric properties (i.e., relative permittivity εr and conductivity δ) of the phantoms were verified with SPEAG microwave probe and the calibrated Vector Network Analyzer (VNA) before being used in the actual measurements with the vest. The dielectric properties of the developed phantoms are included in Table 1 as well. Figure 1 presents the phantoms both for the dense breast model and less dense breast model as well as the final measurement setup with phantoms on a mannequin torso. 

### 2.3. Flexible Antennas and Monitoring Vests

**Figure 2:**
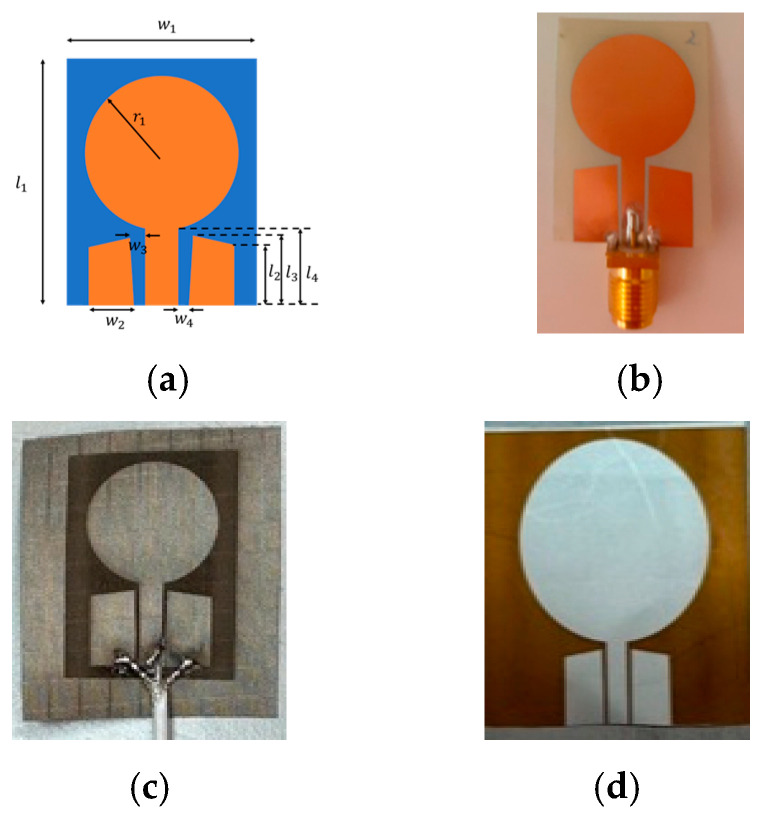
Antennas used in the vest. (**a**) UWB monopole antenna design, (**b**) UWB monopole with flexible laminate substrate, (**c**) UWB monopole with conductive textile material, (**d**) Kapton polyamide substrate-based larger monopole [31].

This Section presents the antennas and tumor monitoring vests utilized in this study. Figure 2a presents the UWB monopole antenna design which acts as a foundation for three fabricated antennas: Antenna1, laminate substrate UWB monopole presented in Figure 2b; Antenna 2, textile UWB monopole (Figure 2c); and Antenna 3, Kapton-based larger monopole (2d). 

Antenna 1 with a flexible laminate substrate was originally conceptualized for the purpose of in-body monitoring and communications within the frequency spectrum of 2–10 GHz in [11]. Such a wide frequency band allows studying optimal frequency ranges for different medical monitoring applications. Its physical measurements are 2 cm by 3 cm; dimension details are given in Table 2.

Antenna 2 represents a textile-adapted version of Antenna 1. The construction process involves the use of laser-assisted etching methods to craft the antenna’s design onto a distinct textile base. Utilizing the LPKF ProtoLaser U3 device, the etching is executed with high precision, facilitating the creation of the antenna pattern with exactitude on the fabric. This precision crafting allows for the antenna’s flawless incorporation into wearable devices and intelligent fabric applications. Maintaining the same blueprint and functional attributes as Antenna 1, Antenna 2 ensures uniformity in performance, while also providing the versatility to be incorporated into a vest.

Antenna 3 is featured with a Kapton polyamide substrate and has slightly larger dimensions, 4 cm by 4 cm, than Antennas 1–2. Antenna 3 includes a more substantial radiator to enhance its radiation characteristics towards the body. The fabrication process of Antenna 3 commences with the selection of a 50 µm flexible Kapton polyamide substrate. This material was chosen for its thermal stability and electrical insulation properties, which are ideal for antenna applications. The initial phase of the procedure entails utilizing an EKRA screen printing apparatus to apply a square motif onto the Kapton substrate. Screen printing, a prevalent method, entails the deposition of ink through a meticulously woven mesh onto the substrate, thereby imprinting the intended design [31]. For this purpose, a bespoke screen featuring an aperture that aligns with the square design was employed. The selected ink for this application was DuPont 5964H silver ink, renowned for its excellent conductive properties, which are essential for the antenna’s functionality. The fabrication of Antenna 3 begins with screen printing, where ink is pressed through a mesh to transfer a square pattern onto Kapton. A screen designed for this pattern and DuPont 5964H silver ink, chosen for its conductivity, are utilized. The substrate is then dried at 80 °C for an hour to ensure ink adhesion. The LPKF ProtoLaser U3 machine performs laser etching, selectively removing ink to reveal the antenna design. Precision in this step is critical for the antenna’s performance. After etching, the antenna is cleaned and inspected to ensure quality, removing any residues or imperfections that could impede functionality. This meticulous process guarantees the antenna’s reliability and efficiency.

**Figure 3:**
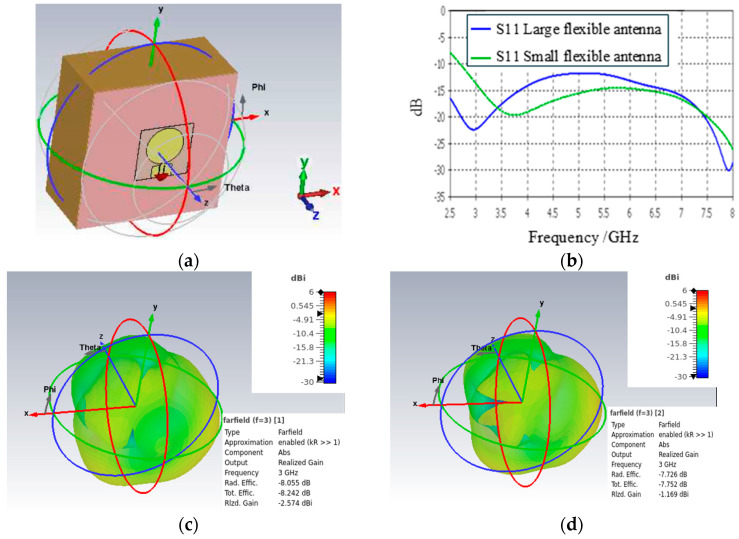

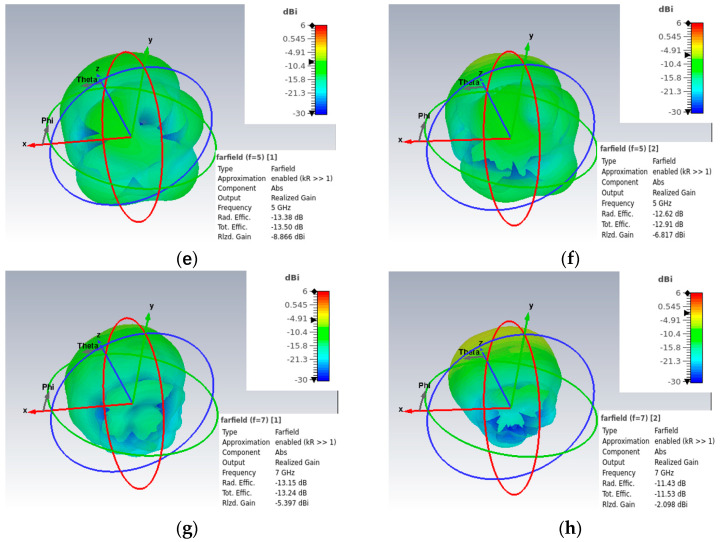
(**a**) Tissue layer model used in antenna characteristics simulations, (**b**) S11 parameters of small and larger flexible antennas, (**c**–**h**) radiation patterns of small flexible antenna (left side of figure) and larger flexible antenna (right side of figure) at 3 GHz, 5 GHz, and 7 GHz.

The dielectric properties of antenna substrates are presented in Table 3. The radiation characteristics of the small and larger flexible antennas are evaluated with simulations using a layer model consisting of skin and fat layers which are the outermost tissue layers in breast, as illustrated in Figure 3a. S11 parameters of both antennas are compared in Figure 3b. It was found that both antennas exhibit good matching in the evaluated frequency range of 2.5–8 GHz. Figure 3c–h illustrate the radiation patterns of the small and larger flexible antennas at 3, 5, and 7 GHz, with the results for the small flexible antenna shown on the left side of the figure and those for the larger flexible antenna on the right side. It should be noted that for the radiation pattern illustration, the layer model in Figure 3a is rotated 180 degrees to better demonstrate the radiation pattern towards the body. This orientation is more meaningful for applications studying tissue changes. Additionally, the figures depict values for the realized gain and efficiency. 

As known from the literature, antennas with a thin and flexible substrate have weaker performance compared to antennas with a rigid substrate [11]. From the results presented in Figure 3c–h, it is found that the larger flexible antenna slightly outperforms the smaller flexible antennas, especially in terms of realized gain. However, the differences in the lobe strengths towards the body are not very significant. Hence, both antenna structures are feasible for a breast tumor monitoring vest. Measured characteristics of these antennas are evaluated in [31].

**Figure 4:**
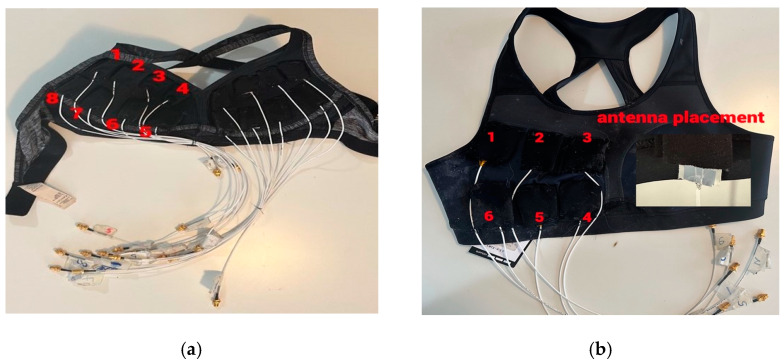
The developed breast tumor monitoring vest types used in the evaluations: (**a**) Vest I with smaller flexible antennas and (**b**) Vest II with larger flexible antennas [31]. The numbers above the antenna pockets indicate the antenna number.

The first monitoring vest, “Vest I”, illustrated in Figure 4a, was made for a sport bra, by sewing eight pockets designed to accommodate Antennas 1–2 (smaller monopoles with flexible laminate and textile versions). The second monitoring vest, “Vest II” shown in Figure 4b, was made with six pockets and intended for use with the larger Kapton substrate-based antennas. The alignment of these pockets is determined based on simulations conducted in [29]. Antennas within the same row were positioned 1.5 cm apart horizontally, and antennas in different rows were placed 2 cm apart vertically in both vests. To connect the antennas to the measurement equipment, thin, SMA straight plug RF cables (P/N:415-0070-MM500 Johnson—Cinch Connectivity) [34] were used. These cables feature SMA connectors on both ends. The flexible antenna was directly soldered to the cut side of the cable. The RG-178 cable used has an impedance of 50 ohms. To ensure stability and minimize mechanical vibration, the openings of the pockets were stitched, and the antennas were securely fixed within the pockets. Vest I is suitable for evaluations for small and medium sized breasts and Vest II for medium and large sized breasts.

### 2.4. Simulations

**Figure 5:**
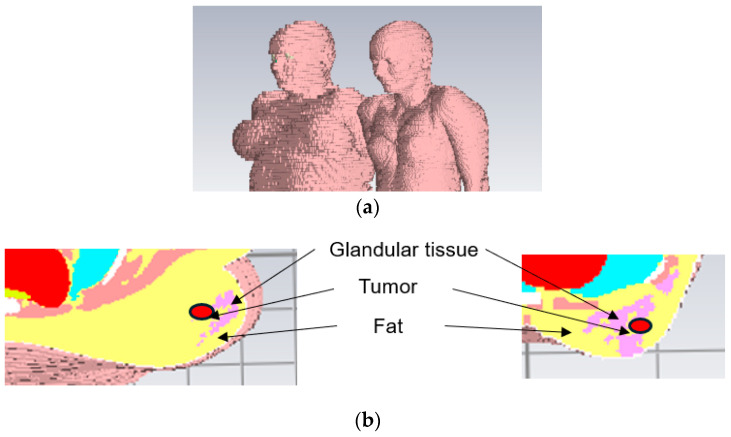
(**a**) Emma (**left**) and Laura (**right**) voxel models used in the simulations, (**b**) cross-section of Emma voxel (scattered fibroglandular tissue, **left**) and cross-section of Laura voxel (heterogeneous glandular breast tissue, **right**).

Simulations were carried out using electromagnetic simulation software CST [33] and its anatomically realistic voxel models Laura and Emma, as shown in Figure 5. The Laura voxel corresponds to a lean woman with breast density type heterogenous consisting of glandular tissue and fat tissue relatively evenly. The Emma voxel resembles an overweight woman having breast density scattered fibro glandular (less dense) in which the breast consists of mostly fat tissue and only slightly glandular tissue. As illustrated in Figure 5, the voxel models exhibit pixelation, and there is a noticeable variation in mesh cell sizes among the different voxel models.

The evaluations for the Emma voxel were carried out with Vest II due to the larger breast size and for the Laura voxel with Vest I. The tumor was set in the middle area of the breast, approximately in the same location as with phantoms. The simulations were conducted in the reference case without any tumor and with tumors having sizes of 1 cm and 2 cm.

## 3. Results and Discussion

In this Section, detectability of breast tumors is evaluated using both vest types and the developed phantoms for dense and less dense breast phantoms. This study examined three scenarios: one without tumors (reference case), one with a 1 cm tumor, and one with a 2 cm tumor. The objective was to understand how tumors affect the channel characteristics between different antenna pairs of the monitoring vest. The measurement-based evaluations were verified with simulations using voxel models. The size and shape of the voxel models and phantoms are not equal, and thus the results are not fully comparable. However, the objective was to determine whether similar trends are observable in both simulations and measurement-based evaluations of tumor detection.

### 3.1. Measurement-Based Evaluations for Vest I and Vest II

Firstly, the experiments were carried out with “Dense” breast phantom and Vest I in which Antenna 1 (flexible laminate substrate) was embedded. In this case, the analysis focuses on evaluating channel transfer functions between antennas 2 and 7 (*Case1a*) as well as antennas 2 and 5 (*Case1b*), i.e., S27 and S25 parameters. These parameters involve the antennas that are nearest to the tumor phantom, thereby exhibiting the most significant disparities in channel characteristics between the tumor and the reference case. 

**Figure 6:**
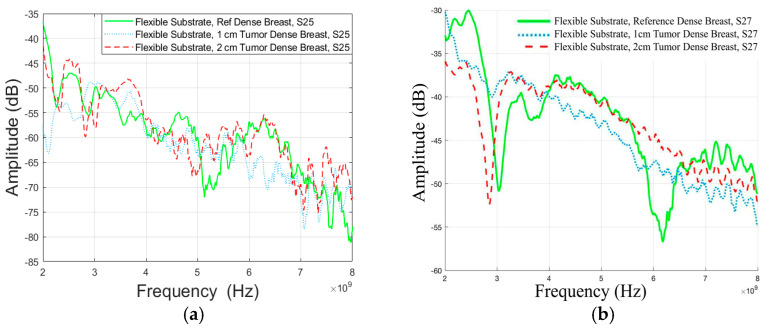
Channel evaluations between (**a**) antennas 2 and 5 (Case 1a) and (**b**) antennas 2 and 7 (Case 1b) for Vest I with Antenna 1 and “Dense” breast phantom.

The S25 parameter results in the reference case and tumor cases presented in Figure 6a. It is observed that the presence of tumors decreases channel attenuation at lower and higher frequencies. The difference between the 1 cm tumor and the reference case is approximately 1.33 dB at 4 GHz and 6.14 dB at 8 GHz. The difference between the 2 cm tumor and the reference case is approximately 1.99 dB at 4 GHz and 8.48 dB at 8 GHz. These changes in channel strengths of reference and tumor cases are attributed to the higher dielectric property values of the tumor compared to the glandular tissue, leading to additional reflections and diffraction as well as higher propagation loss for the signal travelling through the tumor tissue. At most frequencies in this range and with this antenna combination, additional reflections positively affect multipath components arriving at the receiving antenna through or on the breast tissue. With a larger tumor, the impact is also more significant.

In the case of S27 parameters shown in Figure 6b, there was a consistent increase in channel attenuation when tumors are present. The difference between the 1 cm tumor and the reference case was approximately 11.05 dB at 3 GHz and 5.98 dB at 6 GHz. The difference for the 2 cm tumor case was approximately 7.51 dB at 3 GHz and 8.81 dB at 6 GHz. The impact of tumors on channel attenuation was not observed at 4 and 8 GHz, indicating that the detectability of tumor size changes with frequency. These findings suggest that the presence and size of tumors in breast tissue significantly impact channel attenuation, which varies with frequency and the specific antenna combination used. 

**Figure 7:**
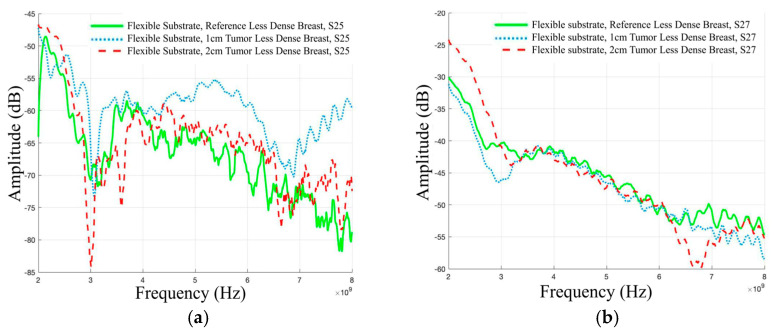
Channel evaluations between the (**a**) antennas 2 and 5 (Case 2a) and (**b**) antennas 2 and 7 (Case 2b) for Vest I with Antenna 1 and “Less Dense” breast phantom.

Next, the evaluations are conducted using the breast phantom type “Less Dense” and Antenna 1, Case 2. The S27 and S25 results (Case 2a and Case 2b, respectively) are shown in Figure 7a,b, respectively. In S27 parameter, both lower and higher frequencies experienced a reduction in attenuation due to tumors. The 1 cm tumor resulted in an attenuation difference of approximately 0.75 dB at 3.5 GHz and 0.39 dB at 6 GHz. Similarly, a 2 cm tumor led to a difference of about 0.65 dB at 3.5 GHz and 1.1 dB at 6 GHz. The differences in channel parameters due to tumors were slightly smaller compared to denser breast types. This is because fat, being a less obstructive medium, allowed the predominant signal components to propagate from fat.

Conversely, when considering channel parameter S25 (Figure 7b), tumors reduced channel attenuation only at higher frequencies. For a 1 cm tumor, there was a decrease of about 0.68 dB at 4 GHz and a substantial 19.18 dB at 8 GHz. In the case of a 2 cm tumor, the difference was approximately −3.28 dB at 4 GHz and 6.35 dB at 8 GHz. The substantial attenuation difference at 8 GHz can be explained by the increased impact of the tumor’s dielectric properties on signal propagation at this higher frequency. The analysis showed a consistent decrease in channel attenuation when tumors were present, particularly at the higher frequency of 8 GHz. While the changes induced by tumors were less pronounced between antennas 2 and 7, they remained detectable, suggesting that tumor detection is feasible even in less dense breast tissues.

**Figure 8:**
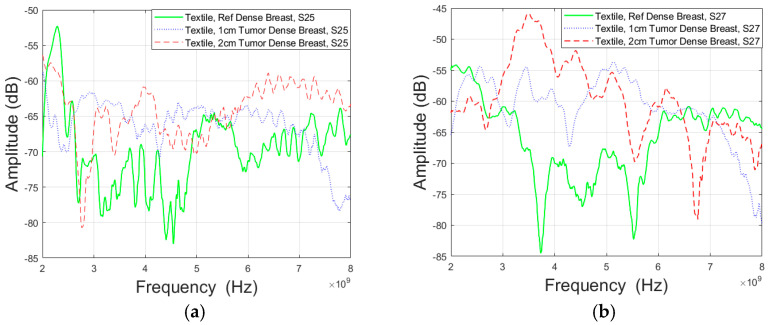
Channel evaluations in Case 3 between the (**a**) antennas 2 and 5 (Case 3a) and (**b**) antennas 2 and 7 (Case 3b) for Vest I with Antenna 2 and “Dense” breast phantom.

The evaluation was further extended to include the use of Vest I with a conductive textile antenna configuration, providing an alternative setup for channel parameter assessments. Experimental investigations using a “Dense” breast phantom and Antenna 2 revealed that the presence of tumors had a substantial effect on channel attenuation at various frequencies and between specific antennas. In Figure 8a, when considering antennas 2 and 5, it was observed that channel attenuation decreased at both higher and lower frequencies when a large tumor was present compared to the reference case. Attenuation difference between 1 cm tumor and the reference case was approximately 9.59 dB at 4 GHz and −8.97 dB at 8 GHz. Similarly, for a 2 cm tumor, the difference was approximately 14.8 dB at 4 GHz and 4.43 dB at 8 GHz.

The presence of tumors also led to a decrease in channel attenuation in the case of S27 parameters, as shown in Figure 8b. In the case of the 2 cm tumor, this reduction was observed at both higher and lower frequencies, whereas for the 1 cm tumor, it was evident only at higher frequencies. Specifically, the difference in attenuation between the 1 cm tumor and the reference case was approximately −0.86 dB at 3 GHz and 4.34 dB at 6 GHz. For the 2 cm tumor case, the difference was approximately 4.96 dB at 3 GHz and 4.38 dB at 6 GHz. The results observed with textile antennas are similar to those with flexible laminate antennas in terms of how tumors affect signal strength when detecting breast tumors. This means that both textile and PCB antennas show consistent results in tumor detection, indicating that these methods are reliable for this purpose. 

**Figure 9:**
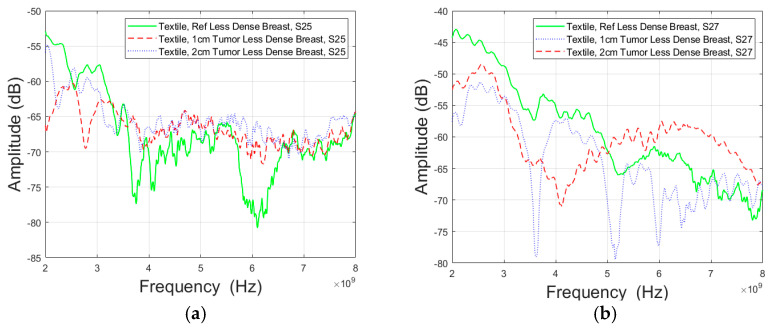
Channel evaluations between in Case 4 (**a**) antennas 2 and 5 (Case 4a) and (**b**) antennas 2 and 7 (Case 4b) for Vest I with Antenna 2 and “Less Dense” breast phantom.

Between the antennas 2 and 5 presented as Case 4a in Figure 9a, the presence of tumors notably reduced channel attenuation, particularly in the frequency range from 3.5 GHz to 6.5 GHz when compared to the reference case. The attenuation difference between a 1 cm tumor and the reference case was around 3.8 dB at 4 GHz and a marginal 0.07 dB at 8 GHz. Similarly, for a 2 cm tumor, the difference was about 1.72 dB at 4 GHz and 0.3 dB at 8 GHz. In Figure 9b, considering antennas 2 and 7 (Case 4b), tumors also resulted in a reduction in channel attenuation, but this effect was primarily observed at 8 GHz. Specifically, the attenuation difference for a 1 cm tumor at 8 GHz was roughly 0.49 dB, while for a 2 cm tumor, it was approximately 1.44 dB at 8 GHz.

The study also examined the performance of Vest II, a system designed for larger breast phantoms (Case 5). Channel parameter evaluations for breast tumor monitoring were conducted using dense breast phantom. The experiments utilized Antenna 3, which is the bigger antenna (monopole 2) constructed with a flexible Kapton polyamide substrate. The primary objective was to analyze the channel characteristics between antennas 1 and 6 as well as antennas 3 and 6, to understand the influence of tumors on channel attenuation in the vicinity of the tumor.

**Figure 10:**
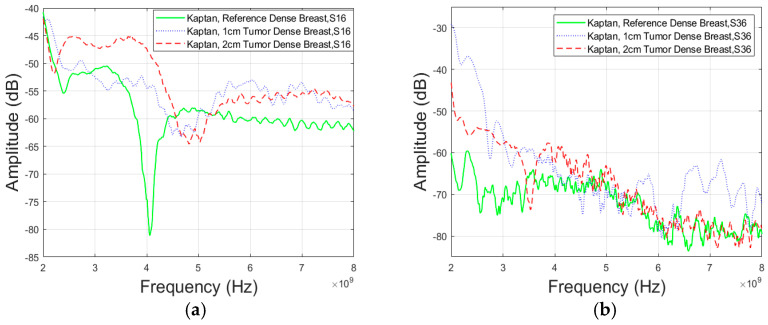
Channel evaluations for Case 5 between the (**a**) antennas 1 and 6 (Case 5a) and (**b**) antennas 3 and 6 (Case 5b) for Vest II with Antenna 3 and “Dense” breast phantom.

For S16 parameter (Case 5a), the difference between the 1 cm tumor and reference case illustrated in Figure 10a, is approximately 3.67 dB at 4 GHz and 20.11 dB at 8 GHz. Also, the difference between the 2 cm tumor and reference case, is approximately 4.5 dB at 4 GHz and 26.89 dB at 8 GHz. Between antennas 3 and 6, (Case 5b) in Figure 10b, the attenuation difference for a 1 cm tumor is 5.59 dB at 4 GHz and 7.18 dB at 8 GHz. For a 2 cm tumor, the attenuation is 7.42 dB at 4 GHz and 1.42 dB at 8 GHz.

**Figure 11:**
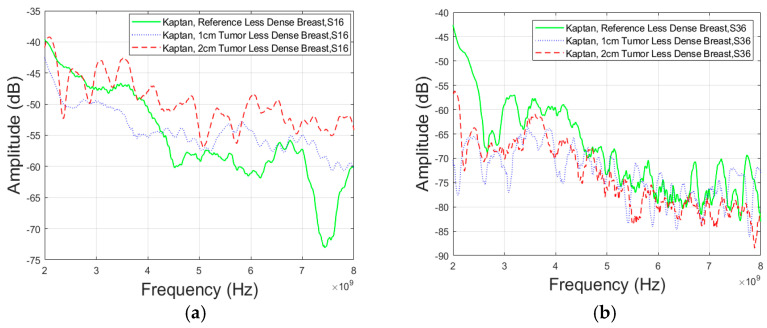
Channel evaluations for Case 6 between (**a**) antennas 1 and 6 (Case 6a) and (**b**) antennas 3 and 6 (Case 6b) for Vest II with Antenna 3 and “Less Dense” breast phantom.

In addition to the previous findings, the study further included the assessment of channel parameters for a less dense breast type phantom using Antenna 3. The channel characteristics between antennas 1 and 6, depicted in Figure 11a (Case 6a), and antennas 3 and 6 in Figure 11b (Case 6b) show no difference between the tumor and reference case at lower frequencies Also, the difference between the 2 cm tumor and reference case is approximately 6.3 dB in Figure 11a and 4.98 dB in Figure 11b at 8 GHz. 

The Vest II system demonstrates a heightened ability to detect tumors, especially in dense breast, highlighting more noticeable differences caused by tumors. These findings highlight the system’s reliability in identifying tumors across varying breast sizes. The study consistently reveals the Vest II’s capability to discern tumor-induced alterations in channel characteristics, with a reduction in channel attenuation when tumors are present. This attenuation change ranges from approximately 0.1 dB to 27 dB, dependent on factors like frequency and tumor size. Tumors introduce additional reflections and diffraction, significantly impacting signal propagation within breast tissue. The results emphasize the potential of tumor detectability and offer valuable insights for the development of effective tumor detection systems. Understanding how tumors affect channel parameters and considering breast tissue characteristics can propel the design of breast tumor monitoring technologies, contributing to early detection, better patient outcomes, and advancements in breast cancer research.

### 3.2. Simulation-Based Evaluations with Voxel Models

Simulations were conducted with the Emma and Laura voxels with tumor sizes 0.5 cm, 1 cm, and 2 cm as well as in the reference case. The Emma voxel was considered to use Vest II with Kapton substrate antennas due to larger breast size and the Laura voxel used Vest I with flexible laminate antennas due to smaller breast size. Similarly to the measurement-based evaluations, channel characteristics were calculated between different antenna pairs, but only the S-parameters related to the antennas which were closest to the tumor are presented for brevity. In the case of the Emma voxel, S26 parameters are presented, and for the Laura voxel, S16 parameters.

**Figure 12:**
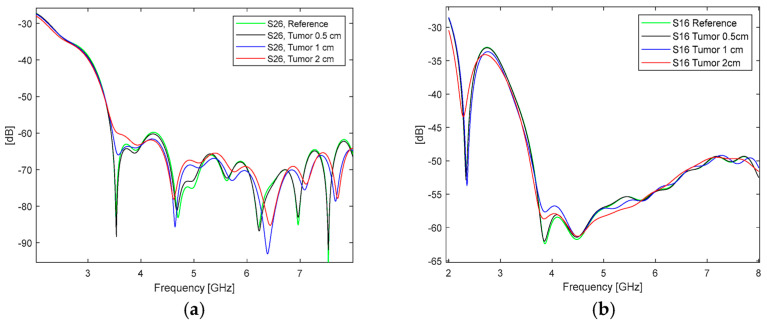
Case 7: Simulation-based channel evaluations with different tumor sizes: (**a**) S26 results using Emma voxel (Case 7a) and (**b**) S16 results using Laura voxel (Case 7b).

The S26 results for the Emma voxel are presented in Figure 12a and the S16 results for the Laura voxel in Figure 12b. Also, the simulation results show that tumors clearly affect the channel characteristics. The differences are larger with the Emma voxel than with the Laura voxel since the Emma voxel has significantly less glandular tissue, which eases tumor detection (the difference between the dielectric properties of tumor and fat tissue is larger than those of tumor and glandular tissue). In particular, detectability of 2 cm tumor tissue is straightforward since it may even cause over 10 dB difference compared to the reference case. Even with the Laura voxel, clear differences, up to 6 dB, can be seen at 2.5 GHz and 3.8 GHz. The simulation results with 0.5 cm tumors are promising as well: although the difference to the reference case is very minor, maximum 0.5 dB in this antenna location, there could be changes to detect such small tumors with more directional antennas. This will be left as our next future work. When comparing simulation and measurement results, one can note similar trends in both cases: the impact of the tumor on the channel characteristics is frequency-dependent and tumor size-dependent. Compared to the measurement results, the differences in channel parameters caused by tumors are smaller. Nevertheless, simulation and measurement results are not aimed to be compared directly since the dimensions and tissue constitutions are different. 

The effects of tumors on channel characteristics are summarized for all study cases (1–7) in Table 4. This table provides insights into which portions of the evaluated 2–8 GHz band exhibit an increase or decrease in the channel parameter, denoted by ‘+’ and ‘−’ respectively. Furthermore, if the trend is inconsistent, i.e., the impact varies significantly across the band, it is indicated by ‘u’. The authors in Ref. [29] presented power flow simulation results demonstrating the impact of tumors on signal propagation within breast tissue. The findings indicate that the tumor’s position relative to the antennas significantly influences whether it increases or decreases channel attenuation. The accompanying Table 4 underscores the variability in how tumors affect different frequencies and scenarios. This variability offers valuable insights into how tumors modify channel characteristics across various frequency ranges. However, a common trend observed is that in the lower part of the evaluated frequency range (2–4 GHz), the presence of a tumor in dense breast tissue increases the channel gain for both presented channel parameters (S25 and S27). Additionally, with textile antennas, in the middle part of the frequency range (4–6 GHz), the tumor impact also increases the channel gain. For less dense breast tissue, a similar trend is observed for the S25 parameters with flexible and textile antennas, and the corresponding S36 parameters with Kapton antennas: the presence of a tumor increases the channel gain at 4–6 GHz and, in most cases, also at 2–4 GHz. Overall, the S25 channel parameters with small flexible and textile antennas, and the S36 parameters with Kapton antennas, are effective for detection as they exhibit logical changes in both dense and less dense breast tissues.

The acquired simulation and measurement results with realistic models are promising: even the small-sized tumors in all breast density types can be detected with the proposed vest type of monitoring device from channel parameters. Such a simple S-parameter analysis-based vest could give an indication to the user on their breast health status and also advice to go for hospital for more accurate diagnostics. The detectability of possible tumors could be further improved with channel analysis methods in different domains [8]. Additionally, measured S-parameters could be utilized for imaging, which however would increase the computational load as well as costs of the device. Thus, imaging-based monitoring vest could be used in healthcare centers for more accurate pre-diagnostics. However, both monitoring vest types could decrease breast cancer mortality by enabling more frequent breast health checks outside the hospitals which is essential especially for the risk groups or people living in rural areas. 

One of the main challenges in this kind of monitoring vest is the development of extensive reference data banks for a wide variety of breast types. Since the breast type (breast density and size) clearly affects the S-parameters, it is crucial that measured data are compared with correct reference data, as discussed in [9]. 

### 3.3. Time-Domain Evaluations

**Figure 13:**
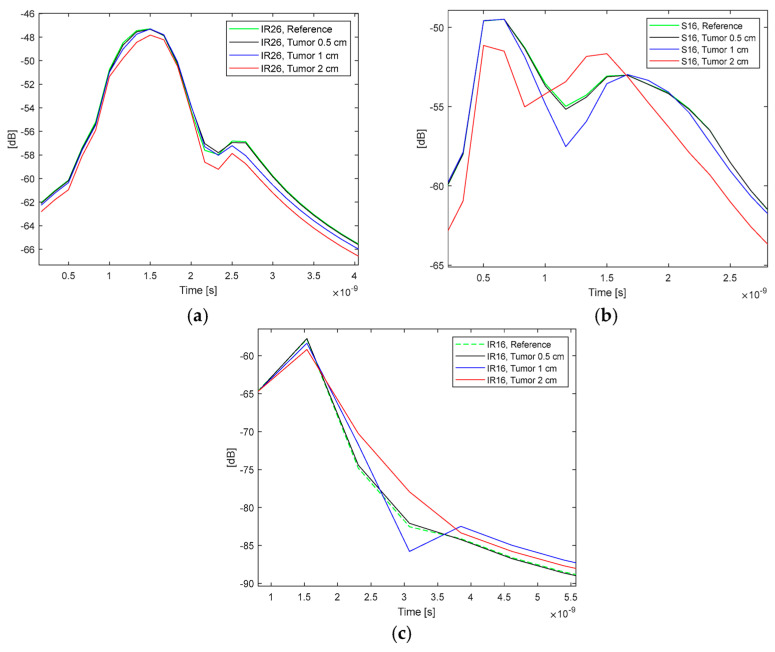
Time-domain channel evaluations with different tumor sizes and different IFFT lengths: (**a**) Impulse response IR26 results using Emma voxel with full band IFFT conversion, (**b**) IR16 results using Laura voxel, with full band IFFT conversion, (**c**) IR16 results using Laura, with IFFT conversion to 4.5–5.8 GHz.

In this subsection, several time-domain evaluations are conducted on both simulation and measurement data. The time-domain conversion is achieved using the Inverse Fast Fourier Transform (IFFT) applied to the complex channel parameter data. In this analysis, the direct IFFT was utilized without any oversampling or filtering. Initially, the IFFT was performed across the entire simulated bandwidth of 2–8 GHz. The resulting impulse responses (IR) are depicted for the Emma and Laura cases in Figure 13a and Figure 13b, respectively.

For the Emma voxel, the trend is particularly evident near the second peak, with even minor variations in the main peak being observable. Conversely, for the Laura voxel, the differences are more pronounced, especially in the presence of a 2 cm tumor. However, these differences are less consistent compared to the Emma case.

As illustrated in Figure 12a,b, the discrepancies between the reference and tumor cases vary across the evaluated frequency band. Therefore, for practical applications, it is more appropriate to select only the frequency range where the changes are consistent for analysis. For instance, in Laura’s case, the frequency domain channel responses exhibit logical changes within the 4.5–5.8 GHz band. Subsequently, the IFFT is applied solely to this range, with the results presented in Figure 13c. As observed, the IR changes are now more consistent.

## 4. Summary and Conclusions

This study outlined the development of an initial prototype for a breast tumor monitoring vest utilizing microwave technology. It details the design of different vests versions incorporating three different flexible antennas, and their assessment using 3D human tissue phantoms that represent different breast densities. Furthermore, the accuracy of the measurement data is verified through electromagnetic simulations conducted on anatomically precise voxel models. The ability of vests to detect breast tumors of different sizes is evaluated by analyzing channel transfer scattering parameters of the antennas embedded in the vest. 

This paper included several novel components which have not been studied in the previous literature: use of different flexible antennas which could be realistic and suitable in practical application, evaluations using realistic phantoms of different sizes and breast densities, and realistic simulation models. Additionally, this is the first paper presenting the detectability of 0.5 cm sized tumors using anatomically realistic simulation models and flexible antennas. Moreover, this is the first monitoring vest study in which cabling of the vest was planned to avoid uncertainties in the measurement results, hence also enabling self-monitoring.

Simulation and measurement evaluations promisingly showed that the proposed vest can detect early-stage tumors as small as 0.5–1 cm in diameter even inside the glandular tissue. It was observed that the detectability of breast tumors depends on the frequency, antenna selection, tumor size, and breast type, resulting in differences of 0.5–30 dB between the tumorous and reference cases. The detectability could be further improved with directional antennas which is left for future work.

The challenge with microwave-based tumor detection is that the differences caused by tumors in channel responses are frequency-dependent: at certain ranges the tumors increase the channel strength and other ranges decrease channel strength. In addition to breast density type, the tumor location and size have clear impacts on the differences, along with antenna type. However, as shown in Table 3, some common trends can be identified where changes are logical. The most effective channel parameters for detection are the S25 parameters with small flexible and textile antennas, and the S36 parameters with Kapton antennas, as they exhibit consistent and logical changes in both dense and less dense breast tissues at same frequency ranges. For a practical scenario of channel analysis-based breast tumor detection, comprehensive reference databases for different breast densities will be required. Additionally, artificial intelligence will be used to recognize a patient’s breast density and select the corresponding reference database and the most effective parameters to maximize detectability of the tumors.

In this paper, we presented only the analysis of S-parameters in reference and tumor cases, as the primary objective was to demonstrate the evaluation results as an initial proof-of-concept for breast tumor monitoring vests using realistic simulation and emulation platforms. A monitoring vest based on S-parameter analysis provides a computationally lower-complexity and cost-efficient solution for breast health checks in smaller healthcare centers or even for home monitoring. The vest could indicate the possible presence of tumors and advise the individual to visit a hospital for more detailed examination.

The acquired S-parameters could be further employed to analyze data across various domains and used to generate images through imaging algorithms [8]. Furthermore, AI-based algorithms can be leveraged to determine whether the breast is healthy or contains tumors, as well as to ascertain the location and type of any tumors. These features could be realized in more advanced vests which, on the other hand, would require more computational resources and would be also less cost-efficient. More detailed analysis of the S-parameters in different domains as well as evaluation of different imaging algorithms will be addressed in subsequent research.

The impact of the breast tumor monitoring vest for healthcare could be significant: it could decrease breast cancer mortality by enabling more frequent breast health checks also outside the hospital and in rural areas providing benefits especially for risk groups. The simpler vest versions, which utilize channel parameter analysis and are therefore more cost-efficient, could be used for home monitoring. These biodevices could provide an indication of when it is necessary to visit a hospital for more accurate examinations. The more advanced vest versions, which can produce images from the S-parameters and which will be evaluated in our future study, could be used in healthcare centers for pre-diagnostics. One of the main challenges for both vest versions, and for microwave-based breast tumor detection in general, lies in developing comprehensive reference data banks for a wide variety of breast types. Creating these data banks requires extensive evaluations using realistic simulation and emulation models, as well as measurements with human subjects.

The proposed monitoring vest operates both at ISM 2.5 GHZ as well as UWB Band 3–10 GHZ which enables smooth integration to existing standardized technologies. The proposed vest could be used as pre-diagnosis method outside the hospital and also as a complimentary screening method for currently used mammography and ultrasound techniques. The vest can be integrated with telemedicine platforms, enabling remote monitoring and diagnosis. This integration is particularly advantageous for patients in remote or underserved areas, granting them access to advanced diagnostic tools.

In the future, breast monitoring vests must be adapted to meet clinical protocols. Before reaching the clinical testing phase, comprehensive studies with realistic models should be conducted to analyze sensitivity and specificity.

## Figures and Tables

**Table 1:** Recipes for preparing breast tissue and tumor phantoms, their measured dielectric properties, and reference values at 6 GHz.

Ingredients	Composition for Different Phantoms				
	Fat	Skin	Tumor	Muscle	Glandular
DI-water	3 mL	12 mL	20.3 mL	10 mL	10.1 mL
Gelatin	2 g	3.6 g	1.6 g	6.0 g	2.0 g
Sunflower Oil	-	2.0 mL	1.1 mL	3.4 mL	-
Dishwasher	0.5 mL	1 mL	0.9 mL	1.7 mL	-
NaCl	-	-	-	16.7 mL	-
Sugar	-	-	-	-	0.2 g
Propylene Glycol	40 mL	-	-	-	-
Xanthum	1 g	-	-	-	-
${\varepsilon_{r,\;phantom},\delta }_{phantom}\;[S/m]$	5.0/0.95	36/5.9	58/7	54/6.8	51/7.6
${\varepsilon_{r,\;ref},\delta }_{ref}$ [S/m]	4.9/0.7	35/4.0	59/6.9	49/5.2	51/6.0

**Table 2:** Dimensions of the three designed antennas.

Parameter	Dimension (mm)								
	$l_{1}$	$l_{2}$	$l_{3}$	$l_{4}$	$r_{1}$	$w_{1}$	$w_{2}$	$w_{3}$	$w_{4}$
Antennas 1–2	30	9.1	10	11	8.1	20	5.7	0.6	0.4
Antenna 3	40					40			

**Table 3:** Dielectric properties of the antenna substrate materials at 5 GHz.

Substrate Material Dielectric Property at 5 GHz	Teflon	Kapton	PCB	Fabric
Relative permittivity	2.1	9.1	10.0	11.0
Loss tangent	0.001	0.004	0.002	0.112

**Table 4:** Impact of the tumor at different frequencies.

Study Cases	Frequencies with Best Detectability [GHz]	Study Cases	Frequencies with Best Detectability [GHz]
**Case 1a:**		**Case 1b**	Lower: 2–3 GHz (−),
Flexible,	Lower: 3.2–4 GHz (+)	Flexible,	3–4 GHz (+)
Dense breast,	Middle: 4.2–5 GHz (−)	Dense breast,	Middle: 5.8–6.5 (+)
S25	Upper: 7–8 GHz (+)	S27	Upper: 6.5–8 (−)
**Case 2a:**		**Case 2b**	
Flexible antenna,	Lower: 2.5–3 GHz (+)	Flexible antenna,	Lower: 2.5–3.5 (+/−)u
Less Dense	Middle: 4–6.5 GHz (+)	Less Dense	Middle: 4–6.5 (−)
S25	Upper: 6.5–8 GHz (+)u	S27	Upper: 6.5–8 (−)
**Case 3a**		**Case 3b**	
Textile	Lower: 2.5–4 GHz (+)	Textile	Lower 3–4 (+)
Dense	Middle: 4–5 GHz (+)	Dense	Middle 4–6 (+)
S25	Upper: 5.5–7.5 GHz (+)	S27	Upper 7–8 (−)u
**Case 4a**		**Case 4b**	
Textile	Lower: 3–4 GHz (+)	Textile	Lower: 2–5 GHz (−)
Less Dense	Middle: 4–6 GHz (+)	Less Dense	Middle: 5–6 GHz (+/−)
S25	Upper: 7–8 GHz (−)u	S27	Upper: 7–8 GHz (+/−)u
**Case 5a**		**Case 5b**	
Kapton	Lower: 2–4 GHz (+)	Kapton	Lower: 2–4 GHz (+)
Dense	Middle: 4.5–5.2 GHz (−)	Less Dense	Middle: 4–6 GHz (+/−)
S36	Upper: 6–8 GHz (+)	S16	Upper: 6–8 GHz (+)
**Case 6a**		**Case 6b**	
Kapton	Lower: 2–4 GHz (+/−)u	Kapton	Lower: 2–4 GHz (−)
Less Dense	Middle: 4–6 GHz (+)	Less Dense	Middle: 4–6 GHz (−)
S36	Upper: 6–8 GHz (+)	S16	Upper: 6–8 GHz (−)
**Case 7a**		**Case 7b**	
Emma voxel	Lower: 3.5–4.5 GHz (+)	Laura voxel	Lower: 3.5–4.5 GHz (+)
Antenna 2	Middle: 4–6.2 GHz (−)	Antenna 1	Middle: 4.5–5.8 GHz (−)
S26	Upper: 7.8–8 GHz (−)	S16	Upper: 6–8 GHz (+)

## Data Availability

The original contributions presented in the study are included in the article, further inquiries can be directed to the corresponding author.
